# Cardiac sympathetic activity in hypertrophic cardiomyopathy and Tako-tsubo cardiomyopathy

**DOI:** 10.1007/s40336-015-0133-z

**Published:** 2015-08-04

**Authors:** Derk O. Verschure, Berthe L. F. van Eck-Smit, G. Aernout Somsen, Hein J. Verberne

**Affiliations:** Department of Nuclear Medicine, Academic Medical Center, University of Amsterdam, P.O. Box 22700, 1100 DE Amsterdam, The Netherlands; Department of Cardiology, Medical Center Alkmaar, Alkmaar, The Netherlands; Cardiology Centers of the Netherlands, Amsterdam, The Netherlands

**Keywords:** Cardiac sympathetic activity, ^123^I-*m*IBG scintigraphy, Tako-tsubo cardiomyopathy, Hypertrophic cardiomyopathy

## Abstract

^123^I-*meta*-iodobenzylguanidine (^123^I-*m*IBG) scintigraphy has been established as an important technique to evaluate cardiac sympathetic function and it has been shown to be of clinical value, especially for the assessment of prognosis, in many cardiac diseases. The majority of ^123^I-*m*IBG scintigraphy studies have focused on patients with cardiac dysfunction due to hypertension, ischemic heart disease, or valvular disease. However less is known about the role of ^123^I-*m*IBG scintigraphy in primary cardiomyopathies. This overview shows the clinical value of ^123^I-*m*IBG scintigraphy in two types of primary cardiomyopathy: The genetic hypertrophic cardiomyopathy (HCM) and the acquired Tako-tsubo cardiomyopathy (TCM). Cardiac sympathetic activity is increased in HCM and correlates to the septal wall thickness and consequently to the LVOT obstruction. Moreover, increased cardiac sympathetic activity correlates with impaired diastolic and systolic LV function. In addition, ^123^I-*m*IBG scintigraphy may be useful for determining the risk of developing congestive heart failure and ventricular tachycardia in these patients. In TCM ^123^I-*m*IBG scintigraphy can be used to assess cardiac sympathetic hyperactivity. In addition, ^123^I-*m*IBG scintigraphy may identify those patients who are prone to TCM recurrence and may help to identify responders to individual (pharmacological) therapy.

## Introduction

The last decades, ^123^I-*meta*-iodobenzylguanidine (^123^I-*m*IBG) scintigraphy has been established as an important technique to evaluate cardiac sympathetic function. *m*IBG is a norepinephrine (NE) analog that has the same presynaptic uptake, storage and release mechanism as NE. Radiolabeling of *m*IBG with ^123^I allows for imaging with gamma-cameras. Cardiac ^123^I-*m*IBG scintigraphy has been shown to be of clinical value, especially for the assessment of prognosis, in many cardiac diseases [1–5]. Both a decreased late heart to mediastinal ratio (late H/M) and an increased myocardial washout rate (WO) of ^123^I-*m*IBG are associated with a poor prognosis. The majority of ^123^I-*m*IBG scintigraphy studies have focused on patients with cardiac dysfunction due to hypertension, ischemic heart disease, or valvular disease. In general, these studies include heterogeneous populations (e.g., ischemic and non-ischemic). However less is known on the role of ^123^I-*m*IBG scintigraphy in primary cardiomyopathies (i.e., cardiomyopathies predominantly involving the myocardium). In this overview, we will discuss the clinical value of ^123^I-*m*IBG scintigraphy in two types of primary cardiomyopathy: The genetic hypertrophic cardiomyopathy (HCM) and the acquired Tako-tsubo cardiomyopathy (TCM).

## Hypertrophic cardiomyopathy

**Fig. 1:**
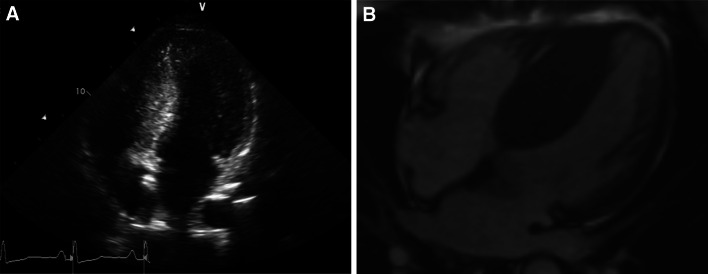
Examples of hypertrophic cardiomyopathy assessed with echocardiography (**a**) and MRI (**b**) showing severe septal hypertrophy of the LV

HCM, the most common of the genetic cardiovascular diseases, is caused by a multitude of mutations in genes encoding for proteins of the cardiac sarcomere. The prevalence of HCM is approximately 1:500 [6]. HCM is defined by a characteristic histopathogical appearance called myocyte disarray [7]. This disarray results in left ventricular hypertrophy with diastolic dysfunction. The prognosis of HCM has an inverse relation to the degree of hypertrophy [8]. Left ventricular outflow tract (LVOT) obstruction is present in 20–25 % of patients with HCM. This obstruction is caused by asymmetric septal myocardial hypertrophy (Fig. 1) [9, 10]. The severity of LVOT obstruction is correlated with impaired excise tolerance, heart failure and sudden cardiac death (SCD). In addition, myocardial fibrosis in HCM is a risk factor for lethal arrhythmias and SCD [11].

### Cardiac sympathetic activity and prognosis in HCM

HCM involves impaired cardiac sympathetic innervation with impaired NE uptake resulting in increased spillover of NE with increased serum levels of NE [12–14]. Since the introduction of cardiac ^123^I-*m*IBG scintigraphy it has been reported that cardiac sympathetic activity in HCM is impaired (i.e., decreased late H/M and increased WO) [15–18]. Some of these studies reported that WO correlates with the severity of hypertrophy [15, 16].

Pace et al. evaluated ^123^I-*m*IBG uptake and WO in relation to left ventricular (LV) function and perfusion [19]. Eleven patients with HCM were enrolled. All patients were subjected to planar and single photon emission computed tomography (SPECT) ^123^I-*m*IBG scintigraphy, ^99m^Tc-MIBI SPECT and echocardiography. WO showed a positive relation with LVOT obstruction (*r* = 0.84, *p* < 0.001) and septum thickness (*r* = 0.76, *p* < 0.01). These data suggest that cardiac sympathetic activity correlates to the degree of septal hypertrophy and consequently LVOT obstruction and diastolic function in HCM. On the other hand, late H/M increases and consequently WO decreases in the months following septal ablation, a percutaneous procedure to induce a septal infarction which results in a reduction of LVOT obstruction [20].

LV function is usually normal at the onset of HCM. However, late in the disease dilatation and LV dysfunction can occur. In end-stage HCM LV dysfunction is related to thinning of the septal wall [21]. This septal thinning is histopathologically associated with extensive myocardial fibrosis [22]. Terai et al. studied the changes of WO from early to end-stage HCM. In this study 46 patients with HCM were enrolled and divided in three different stages of HCM: preserved LV function, dilated LV with preserved LV function and dilated LV with impaired LV function) [23]. Interestingly, regional early uptake was significantly reduced in the septal wall, predominately in the end-stage HCM. In addition, regional washout rate in this group was significantly increased in the apex and lateral wall. These findings could indicate that the development of LV systolic dysfunction caused by myocyte death and increased fibrosis in het septal wall leads to an increase cardiac sympathetic activity resulting in a further increase of WO. Decreased number of myocytes and increased fibrosis result in decreased early uptake of ^123^I-*m*IBG in the septal wall. In response to impaired cardiac function, cardiac sympathetic activity in het apex and lateral wall with viable myocytes increases to preserve LV function.

In line with this study Matsuo et al. reported that in 59 patients with HCM the brain natriuretic peptide (BNP), a marker of LV dysfunction or damage, and left ventricular mass index (LVMI) measured with echocardiography, were independent predictors of late H/M (*p* = 0.0001 and *p* = 0.0009, respectively) [24]. In addition, NE serum level was an independent predictor of WO (*p* = 0.018). The negative correlation between late H/M and BNP (*r* = −0.44, *p* < 0.001), indicates that cardiac sympathetic neuronal dysfunction corresponds to the severity of HCM.

In patients with HCM, congestive heart failure (CHF) caused by LV dilatation and dysfunction is an important determent as well as a predictor of SCD [25–27]. However, clinical tools for predicting the onset of CHF in HCM are limited. Hiase et al. demonstrated that cardiac sympathetic activity could be useful in predicting the onset of CHF in patients with HCM [28]. In this study 84 patients with HCM were enrolled. The prevalence of CHF during a follow-up of 9–86 months was 0 % in patients with H/M >2.11, 3.3 % in patients with H/M, 1.86 to <2.11 and 55.0 % in patients with H/M <1.86. Multivariate analysis showed that cardiac sympathetic activity, as reflected by the late H/M and LV fractional shortening were significant predictors of CHF in HCM.

Since HCM is caused by various gene mutations, each mutation may give rise to a specific pathophysiological pathway which may also result in specific modulations of the sympathetic nervous system. In HCM caused by the Asp175Asn substitution of the alpha-tropomyosin gene (TPM1-Asp175Asn) there is a correlation between WO and LV hypertrophy measured by MRI (*r* = 0.512, *p* = 0.018) [29]. Although these results are in line with the previous reported HCM population, it is unclear if these findings apply to other HCM gene mutations.

### Cardiac sympathetic activity and arrhythmias in HCM

SCD is a serious complication of HCM and is the result of malignant ventricular arrhythmias [i.e., ventricular tachycardia’s (VT) and ventricular fibrillation (VF)] [8, 30, 31]. Most often VT is caused by re-entry mechanisms and occurs in the presence of heterogeneous myocardial excitation [32, 33]. Terai et al. showed that the occurrence of malignant VT in HCM is associated with global cardiac sympathetic activity rather than the heterogeneity of this cardiac sympathetic activity [34]. In this study 44 HCM patients were monitored with ambulatory electrocardiographic monitoring and were assessed with planar and SPECT ^123^I-*m*IBG scintigraphy. 15 patients developed VT, defined as a run of 3 or more consecutive beats at a rate of ≥120 beats/min. WO was significantly increased in the group with VT compared to those without VT (0.27 ± 0.06 vs. 0.17 ± 0.06, *p* < 0.0001). Late H/M and regional ^123^I-*m*IBG parameters from the SPECT images showed no statistically significant difference between the two groups. Although the left ventricular ejection function (LVEF) in patients with VT was decreased compared with those without VT, multiple regression analysis demonstrated that WO was het most powerful predictor of occurrence of VT. The findings of this study suggest that a global rather than regional increase of adrenergic drive (i.e., WO) may increase the heterogeneous conduction of excitation and may easily cause VT in patient with HCM. In addition, in patients with VT the cardiac sympathetic activity is increased probably in response to the impaired LV dysfunction.

### Pathophysiology

Progression of many clinical features of HCM could be explained by stimulation of increased cardiac sympathetic activity. NE induces myocardial cell growth, disarray and scarring [35–37]. Second, α-adrenergic coronary constriction caused by increased NE levels can induce myocardial ischemia [38]. Third, NE increases rate of spontaneous depolarization of myocardial cells, which may predispose to ventricular arrhythmias. Fourth, most HCM patients have an increased global LVEF, indicating increased contractility, which might be due to accelerated cardiac adrenergic activity. Finally, β-adrenergic blocking agents have shown to be efficient in controlling symptoms in HCM [9].

Studies discussed in the previous section (Table 1) are limited by their single center design with differences in acquisition technique (gamma camera, collimator, acquisition time) and semi-quantitative analysis. These differences have hampered multicentre comparison of the ^123^I-*m*IBG scintigraphy. The proposal by Flotats et al. to standardize cardiac sympathetic imaging for ^123^I-*m*IBG scintigraphy will most likely reduce the interinstitutional variation [39]. To reduce the interinstitutional variation Nakajima et al. used a cross calibration phantom method, to convert institutional H/M to standardized H/M [40].

**Table 1:** An overview of the different cardiac ^123^I-*m*IBG scintigraphy studies in HCM with the primary study outcome

References	Patients	^123^I-*m*IBG parameters and morphology
Pace et al. [19]	HCM (*n* = 11)	Late H/M correlates with septal hypertrophy
		WO correlates with LVOT obstruction
Teria et al. [23]	HCM (*n* = 46) with 3 categories: normal LVF, dilated LV, dilated LV with impaired LVF	WO correlates with impaired LV function in HCM
Matsuo et al. [24]	HCM (*n* = 59) and controls (*n* = 23)	Late H/M correlates with BNP and LV mass
		WO correlated with plasma NE levels

References	Patients	^123^I-*m*IBG parameters and prognosis
Hiase et al. [28]	HCM (*n* = 84) and controls (*n* = 18) with three categories late H/M: <2.11 (*n* = 34), 1.86–2.11 (*n* = 30), <1.86 (*n* = 20)	Late H/M independent predictor of onset heart failure
Teria et al. [34]	HCM (*n* = 44) with categories VT (*n* = 15) and no VT (*n* = 29)	Increased WO independent predictor of ventricular tachycardia

## Tako-tsubo cardiomyopathy

**Fig. 2:**
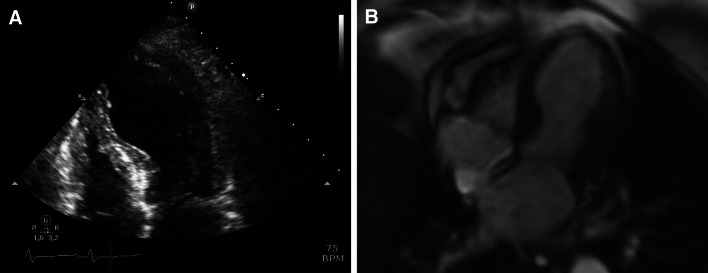
Examples of Tako-tsubo cardiomyopathy assessed with echocardiography (**a**) and MRI (**b**) showing typical apical ballooning with hyperkinesia of the basal segments and dyskinesia of the apical segments of the LV

TCM, also known as stress-induced cardiomyopathy, apical ballooning syndrome or broken heart syndrome was first described in Japan in 1990 [41]. It is characterized by transient systolic dysfunction of apical and/or mid segments accompanied with ballooning of these segments. Importantly, most often wall motion abnormalities extent beyond the distribution of any single coronary artery. The clinical presentation can mimic acute myocardial infarction, in the absence of obstructive coronary artery disease. The Japanese phrase ‘tako-tsubo’ can be translated in English as ‘octopus pot’, a fishing jar with a narrow neck and wide base used to trap an octopus. This description reflects the visual appearance of the heart on left ventriculography or echocardiography (Fig. 2). Considerable evidence points to epinephrine as an important factor in the pathophysiology [42]. In the acute phase of TCM, plasma epinephrine levels are more elevated compared with the acute phase of a myocardial infarction [43].

TCM affects predominantly post-menopausal women and is usually preceded by exposure to physical or emotional stress. Major symptoms of TCM are chest pain at rest, mimicking acute myocardial infarction and dyspnea. In general TCM has a favorable prognosis and after the acute phase left ventricular function normalizes in four weeks [43]. The annual recurrent rate of TCM is 1–2 % [44].

### Tako-tsubo cardiomyopathy and cardiac sympathetic activity

In the sub-acute phase of TCM ^123^I-*m*IBG scintigraphy reveals impaired apical myocardial uptake of ^123^I-*m*IBG on planar images [45]. This is confirmed by SPECT ^123^I-*m*IBG imaging, which demonstrated mainly decreased ^123^I-*m*IBG uptake of the myocardial apex, which correlates with the impaired LV segments [46, 47]. Akashi et al. confirmed these findings in 8 TCM patients using both planar and SPECT ^123^I-*m*IBG imaging [48]. Interestingly, 3 months after the onset of TCM the impaired late H/M was significantly increased (1.89 ± 0.25 vs. 2.16 ± 0.24, *p* < 0.05). In addition, the WO was significantly decreased (0.39 ± 0.10 vs. 0.25 ± 0.06, *p* < 0.05). The SPECT imaging shows impaired uptake of ^123^I-*m*IBG mainly in the apex and inferior wall both in the sub-acute phase as 3 months after the onset of TCM. These results suggest that TCM could be caused by neurogenic myocardial stunning. Recently, we have reported a possible explanation of impaired regional ^123^I-*m*IBG uptake in TCM [49]. The pattern of impaired ^123^I-*m*IBG uptake follows the increasing β_2_AR:β_1_AR ratio from the base to the apex [42, 50]. In addition to the classical apical ballooning TCM there are several case reports of mid-ventricular ballooning, probably caused by a variation of β_2_AR:β_1_AR ratio. Interestingly, it has been reported that in mid-ventricular ballooning SPECT ^123^I-*m*IBG imaging showed impaired uptake of ^123^I-*m*IBG in the mid wall. This underlines our hypothesis that neurogenic stunning occurs in myocardium with increased β_2_AR:β_1_AR ratio.

Although left ventricular function and epinephrine levels are normalized after a few weeks, several case reports show a persistent decrease in ^123^I-*m*IBG uptake on SPECT images in the apical myocardium [46, 51]. The mechanism of this persisting regional impaired ^123^I-*m*IBG uptake is yet unclear. We assume that the increased apical density and sensitivity of the β_2_AR to epinephrine causes a prolonged effect of downregulation of β_2_AR and impaired uptake-1 function (i.e., norepinephrine re-uptake transporter). This causes relatively high levels of epinephrine and NE in the synaptic cleft and may in turn cause these receptors and transporters to recover more slowly compared to more basal located β_2_ARs. In addition, the phenomenon of persisting decreased myocardial ^123^I-*m*IBG uptake may in part be explained by pre-existing myocardial sympathetic denervation. Of interest is whether especially the slow recovery of apical ^123^I-*m*IBG uptake may identify those patients who are at an increased risk of the recurrent TCM.

In conclusion, cardiac ^123^I-*m*IBG scintigraphy can be used to assess cardiac sympathetic activity in primary cardiomyopathies like HCM and TCM.

Cardiac sympathetic activity is increased in HCM and correlates to the septal wall thickness and consequently to the LVOT obstruction. Moreover, increased cardiac sympathetic activity correlates with impaired diastolic and systolic LV function and increased BNP, which is in line with CHF studies [52]. This suggests that increased cardiac sympathetic activity is a result of the unfavorable hemodynamics in HCM. In addition, cardiac ^123^I-*m*IBG scintigraphy may be useful for determining the risk of developing congestive heart failure and ventricular tachycardia in these patients.

^123^I-*m*IBG scintigraphy can be used to assess cardiac sympathetic hyperactivity in TCM patients who are characterized by neurogenic myocardial stunning. In addition, ^123^I-*m*IBG scintigraphy may identify those patients who are prone to TCM recurrence and may help to identify responders to individual (pharmacological) therapy.
